# Lessons From the Communication Bridge Trials: Advancing Meaningful Dyadic Interventions Toward Implementation

**DOI:** 10.1044/2026_JSLHR-26-00074

**Published:** 2026-09-08

**Authors:** Emily Rogalski, Ollie Fegter, Alfred Rademaker, Matthew Bona, Angela C. Roberts

**Affiliations:** a Healthy Aging & Alzheimer's Research Care (HAARC) Center, Biological Sciences Division, University of Chicago, IL; b Department of Neurology, Biological Sciences Division, University of Chicago, IL; c Department of Psychiatry and Behavioral Sciences, Northwestern University Feinberg School of Medicine, Chicago, IL; d Department of Computer Science, Western University, London, Ontario, Canada; e School of Communication Sciences and Disorders, Western University, London, Ontario, Canada

## Abstract

**Purpose:**

This review describes the historical motivations, development, and evolution of the Communication Bridge (CB) research program, which is designed to maximize quality of life and minimize burden for individuals living with dementia and their communication partners. The CB trials described comprise a series of dyadic, nonpharmacologic interventions supporting individuals with primary progressive aphasia (PPA) and their communication partners. Across trials, the CB intervention integrates speech-language, educational, and psychosocial approaches to maximize communication participation, confidence, and self-efficacy.

**Method:**

The CB trials are contextualized within the National Institutes of Health (NIH) Stage Model for Behavioral Intervention Development and the Readiness Assessment for Pragmatic Trials (RAPT) framework to illustrate a systematic pathway from feasibility to implementation readiness. Three sequential telehealth-delivered trials are described: CB1, a pilot feasibility study; CB2, an international randomized controlled trial (RCT); and CB3, an ongoing international RCT designed with clinical practice-aligned procedures to advance readiness for future implementation testing.

**Results:**

Application of the NIH Stage Model and the RAPT framework highlights how staged intervention development can support rigor, reproducibility, and implementation readiness in behavioral intervention research for neurodegenerative conditions. Across trials, person-centered outcomes demonstrated meaningful benefit and maintenance over time, while iterative design refinements addressed feasibility, fidelity, acceptability, and scalability.

**Conclusions:**

The CB research program illustrates how implementation science frameworks can be applied to advance meaningful, dyadic interventions for PPA toward real-world use. Lessons from early-phase trials underscore the importance of person-centered outcomes, staged rigor, and early attention to implementation considerations, with implications for future PPA intervention research and broader speech-language clinical trials.

Primary progressive aphasia (PPA) is a clinical neurodegenerative dementia syndrome characterized by progressive language impairment with relative sparing of other cognitive domains. Clinically, it is differentiated from sudden language deficits due to stroke, and from progressive amnestic syndromes (e.g., Alzheimer's disease dementia [ADD]), where memory is the primary and presenting domain affected (Gorno-Tempini et al., 2011; Jones et al., 2024; Mesulam, Rogalski, et al., 2014). There is phenotypic heterogeneity in PPA that has been used to define three research subtypes or variants: agrammatic, semantic, and logopenic, based on relative impairment and preservation of grammar, fluency, anomia, repetition, and word comprehension. However, these subtypes do not account for the heterogeneity of clinical presentations and are best suited designations when symptoms are neither too mild nor too severe (Jones et al., 2024; Mesulam et al., 2009, 2012; Rogalski & Mesulam, 2009). Neuroanatomically, there is progressive degeneration of the (typically left hemisphere) language network, which is associated with distinct patterns by PPA variant (Gorno-Tempini et al., 2011; Rogalski et al., 2011). There is no single neuropathology responsible for PPA; instead, there are three primary families of proteinopathy that account for PPA: (a) Alzheimer's disease neuropathologic change accounts for 40% of PPA, (b) abnormalities in transactive response DNA-binding protein 43 (TDP-43) explain approximately 30%, (c) and 30% fall under the frontotemporal lobar degeneration spectrum due to four-repeat tauopathies (corticobasal degeneration, progressive supranuclear palsy) or three-repeat tauopathy (Pick's disease; Jones et al., 2024; Mesulam, Rogalski, et al., 2014; Mesulam, Weintraub, et al., 2014). There is no one-to-one correspondence between clinical phenotype and underlying pathology, although probabilistic relationships exist (Jones et al., 2024). The historical absence and current limitations of in vivo biomarkers, as well as the relative rarity of the syndrome, create barriers to the development of interventions and trials for PPA.

PPA is relatively uncommon; it has been historically underrecognized by clinicians, leading to diagnostic gaps. It is not unusual for the diagnostic process to take years and involve several clinicians, at times requiring travel outside of their local region. Diagnostic recognition has improved over the past 20 years due in part to broader recognition of Alzheimer's disease and related disorders, the National Alzheimer's Project Act, the development and maturity of the nongovernmental organizations such as the Association of Frontotemporal Degeneration, the establishment of the International Society for Frontotemporal Dementias, specialized professional interest areas through the Alzheimer's Association, commissioned groups through the Academy of Neurologic Communication Disorders and Sciences, and other efforts. The diagnostic challenges and relative rarity have made it difficult to obtain accurate epidemiologic data on PPA. Some variants of PPA are included under the clinical umbrella of frontotemporal dementias, which have approximate prevalence estimates of 3–15 per 100,000 persons in the United States (Harvey et al., 2003; Ratnavalli et al., 2002). However, comprehensive epidemiologic data remain limited.

PPA is considered a younger onset dementia as onset tends to be < 65 years of age, which creates unique psychosocial and economic challenges (Galvin et al., 2017; Medina & Weintraub, 2007). Individuals with PPA have a higher likelihood of being in the prime-earning phase of their careers and may have dependent children at home, leading to economic burden estimates that are two times higher than ADD, as well as significant negative impacts on health-related quality of life (Galvin et al., 2017; Hopkins et al., 2024; Mesulam, 2003; Nickels & Croot, 2014). Survival is variable, averaging 7–10 years (Diehl-Schmid et al., 2007), though reports of 20 years exist (Dickerson, 2011), emphasizing the relatively long period of time over which care is required. Because of associated functional impairments, the U.S. Social Security Administration includes PPA on its Compassionate Allowances list, which facilitates expedited adjudication of disability claims for eligible applicants. Clearly, the economic, social, and psychosocial impacts of this disease are substantial.

Language loss in PPA disrupts communication, contributes to social isolation, and limits participation in everyday activities, resulting in reduced quality of life and overall well-being (Moeller et al., 2021). In the absence of a definitive cure, nonpharmacologic interventions remain the primary approach to care, with speech-language pathologists most commonly delivering these interventions. There has been substantial growth in PPA-related intervention studies over the last 20 years. Based on a targeted review of the published literature, from 2000 to 2025, there has been a more than ninefold increase in publications on PPA-related intervention.

This review describes the historical landscape, motivations, and evolution of the Communication Bridge (CB) research program, which focuses on maximizing communication participation and quality of life as well as minimizing burden for individuals living with dementia and their communication partners. The CB interventions focused on using speech-language, educational, and psychosocial approaches to maximize communication participation and confidence and self-efficacy, thereby optimizing quality of life for those living with a diagnosis of PPA and their communication partners. The trials are contextualized within the National Institutes of Health (NIH) Stage Model and the Readiness Assessment for Pragmatic Trials (RAPT), highlighting an unmet need to increase opportunities for trials to move toward implementation (Baier et al., 2018, 2019; Onken et al., 2014).

## CB Trial Origins: A Call to Action From Families With Lived Experience

The CB research program emerged from more than a decade of clinical, observational, and community-engaged work with individuals living with PPA and their families. In the early 2010s, Rogalski and colleagues were leading longitudinal, NIH-funded observational studies aimed at characterizing the clinical, neuroanatomical, and biomarker features of PPA, including longitudinal trajectories and associations with emerging in vivo markers such as amyloid positron emission tomography imaging. In parallel, this work was complemented by sustained engagement with individuals with PPA and their communication partners through educational conferences, support groups, and clinician training efforts (Banks et al., 2006; Morhardt et al., 2019; Rogalski et al., 2010; Wieneke et al., 2012). Collectively, these activities were motivated by a shared goal: to improve diagnostic recognition, clinical care, education, and quality of life for individuals living with PPA across the disease continuum.

At the time, the PPA intervention literature was budding and largely characterized by single-case and small case-series designs, with a primary focus on impairment-based language outcomes (Henry et al., 2008; Jokel et al., 2010; Jokel et al., 2002, 2006). These pioneering studies were critical in establishing the feasibility of speech-language intervention in a progressive neurodegenerative condition. However, feedback from individuals with PPA and their families consistently highlighted unmet needs, including access to care and support beyond isolated language impairments, including participation in everyday activities, maintenance of social roles, and reduction of caregiver burden. The CB research program was explicitly designed to build on early feasibility work while broadening the intervention focus to integrate impairment-based as well as participation-centered goals, grounded in the priorities of individuals with PPA and their communication partners.

Despite growing recognition that speech-language pathologists could play a meaningful role in supporting individuals with PPA, families repeatedly reported difficulty identifying clinicians with knowledge of the syndrome or confidence in providing intervention (Croot, 2018; Kortte & Rogalski, 2013; Rogalski & Mesulam, 2009; Rogalski et al., 2016; Taylor-Rubin et al., 2022). Therapeutic nihilism, rooted in the progressive nature of the disease and limited training opportunities, emerged as a persistent barrier (Croot, 2018; Kortte & Rogalski, 2013; Nickels & Croot, 2009). These challenges were compounded by the relative rarity of PPA, resulting in significant geographic inequities in access to specialized care.

Concurrently, advances in consumer-grade video communication technologies (e.g., Skype, FaceTime) were transforming how families connected across distance. For those with PPA, video chat also provided the potential to use multimodal communication strategies beyond those available via telephone alone. These developments created a timely opportunity to reconsider traditional, clinic-based models of service delivery and to explore telehealth as a means of addressing geographic barriers, paving the way for larger-scale evidence-based trials. Early philanthropic support and pilot funding enabled the initial development of the CB web application and the launch of a preliminary feasibility study (CB1) evaluating the delivery of intervention via a web-based platform. Such nongovernmental funding mechanisms played a critical role in supporting early-stage intervention development during a period when the program was not yet positioned for NIH funding.

## Systematic Development of the CB Intervention Within the NIH Stage Model

Until recently, speech-language interventions for PPA were supported largely by early-stage studies, limiting evidence-based clinical selection and large-scale implementation. To advance the evidence base toward broader clinical implementation, the CB research program was designed to systematically progress through established stages of behavioral intervention development. Progression from CB1 (Phase 1, Stage I) to CB2 (Phase 2a, Stage II), and now CB3 (Phase 2b, Stage II), illustrates the intentional evolution of the CB intervention within the NIH Phase and Stage Model (Onken et al., 2014).

Briefly, the NIH has two established models for clinical trial and behavioral intervention development. The traditional clinical trial framework is primarily used in drug and device development and consists of four phases that progress with the following focus at each stage: (a) safety and dosage in small groups, (b) efficacy and side effects in larger groups, (c) large-scale effectiveness compared to standard treatment options, and (d) post-marketing surveillance. The NIH Stage Model for Behavioral Intervention Development shares key features with the traditional drug and device development framework but is tailored to behavioral and psychosocial interventions. In brief, Stage 1 focuses on intervention development, feasibility, and pilot testing; Stage 2 focuses on efficacy testing in controlled research settings with high internal validity; and Stages 3–5 address effectiveness, scalability, and dissemination in real-world clinical contexts. Both the phase and stage models are preceded by basic research on mechanisms and intervention targets. Importantly, the Stage Model emphasizes iterative refinement and explicitly encourages consideration of implementation factors early in the intervention development process to maximize the potential for real-world impact on health and well-being.

Across trials, the CB intervention retained several core features while evolving in structure and rigor over time. The intervention was delivered in a dyadic format involving the person with PPA and a communication partner and combined synchronous telehealth sessions with asynchronous home practice through a web-based platform. Core therapeutic elements included individualized goal setting, strategy development, guided asynchronous practice, and communication partner engagement, while the number of sessions, level of standardization, control condition design, and outcome assessment procedures were refined across CB1, CB2, and CB3 in accordance with stage-specific trial aims.

CB1 was launched as a Phase 1, Stage I pilot trial and demonstrated high feasibility of delivering person-centered speech-language therapy through synchronous (live videoconferencing sessions) and asynchronous (web-based home exercises) modalities (Rogalski et al., 2016). The pilot intervention consisted of one intervention block of eight sessions and emphasized individualized communication goals, strategy use in everyday contexts, and active communication partner involvement. An initial published report, of 34 enrolled participants from 21 states and Canada, showed high feasibility with three discontinuations and documented improvements in communication confidence following intervention and maintenance of gains at 6-month follow-up (Rogalski et al., 2016). Importantly, CB1 also identified communication partner engagement as a relevant factor in intervention response (Rogalski et al., 2022). After the initial report, additional participants enrolled, bringing the total to 57 individuals from 26 U.S. states, Canada, Spain, and Singapore, highlighting the potential for international delivery to overcome geographic barriers to care. Feasibility remained high, with fewer than 10% of participants discontinuing the trial.

Building on these findings, CB2 (Phase 2a, Stage II; NCT03371706) was designed to examine intervention dosing, preliminary efficacy, and safety, representing a necessary step in moving beyond feasibility toward efficacy testing in a progressive neurodegenerative population using a randomized controlled trial (RCT) design. CB2 retained the dyadic, goal-based telehealth model while increasing intervention standardization, formalizing treatment dosing across two intervention blocks, and incorporating a matched active control condition. Enrollment was international, including 95 participants from four countries, one U.S. territory, three Canadian provinces, and 25 U.S. states. Attrition remained low at 6%. To align with the rigor typically expected in pharmacologic trials, CB2 incorporated participant blinding, an active control arm, and systematic fidelity monitoring. Outcomes were person centered and clinically meaningful, and were assessed pre- and post-intervention across two intervention blocks, as well as at 12 months post-enrollment to evaluate maintenance of treatment effects. CB2 demonstrated efficacy at the goal level, with participants achieving meaningful improvements on individualized communication goals using Goal Attainment Scaling, supporting both short-term benefit and maintenance over time (Rogalski et al., 2025).

The ongoing CB3 trial (Phase 2b, Stage II; NCT06191198) aligns with the goals of Phase 2b research by evaluating efficacy and safety in a larger cohort (*n* = 100) using an RCT design. CB3 maintains the core dyadic, goal-based telehealth intervention while incorporating refinements informed by CB2, including increased dose within blocks, more robust psychosocial education and support through social work integration, broader enrollment across more diverse real-world settings and clinicians, and exploratory wearable sensor-based outcomes. In addition, CB3 includes planned cost analyses and further shifts selected procedures from the research team toward workflows that more closely approximate routine clinical practice. These refinements are intended to move CB3 closer to intervention delivery procedures consistent with real-world clinical practice (e.g., scheduling logistics, documentation standards, and outcome selection), while preserving essential experimental controls and fidelity monitoring required to establish efficacy.

## Intentional Plans for Implementation Guided by RAPT

Importantly, CB3 was designed with an explicit focus on advancing implementation readiness and positioning the intervention for future embedded pragmatic trials. Accordingly, the CB3 trial framework is informed by the RAPT tool, which was developed to address the recognized need for a systematic and rigorous approach to shaping interventions for successful pragmatic clinical trials (PCTs; Baier et al., 2018, 2019). The RAPT tool encompasses nine domains: implementation protocol, evidence, risk, feasibility, measurement, cost, acceptability, alignment, and impact. Researchers qualitatively assess each domain using published scoring guidance and may use graphical summaries to provide a visual overview of an intervention's strengths and weaknesses.

RAPT ratings were assigned qualitatively using the published RAPT domains and accompanying scoring guidance. For each domain, CB2 was rated qualitatively based on published trial characteristics and outcomes, whereas CB3 ratings were projected based on planned design modifications intended to improve pragmatic readiness. Figure 1 presents RAPT domain ratings for CB2 and projected ratings for CB3, illustrating how the CB3 design further optimizes the CB intervention for pragmatic testing. Upon completion of CB2, only the Risk domain achieved high readiness, supported by established safety data with few adverse events. In addition, all experimental arm participants who completed the trial showed meaningful improvement toward individualized communication participation goals or maintenance of baseline functioning 12 months post-intervention. Most other domains were rated at medium readiness, reflecting the need for continued development and optimization. The Cost domain was not formally assessed in CB2 and therefore remained at low readiness, highlighting a gap to be addressed before pragmatic implementation.

**Figure 1.**
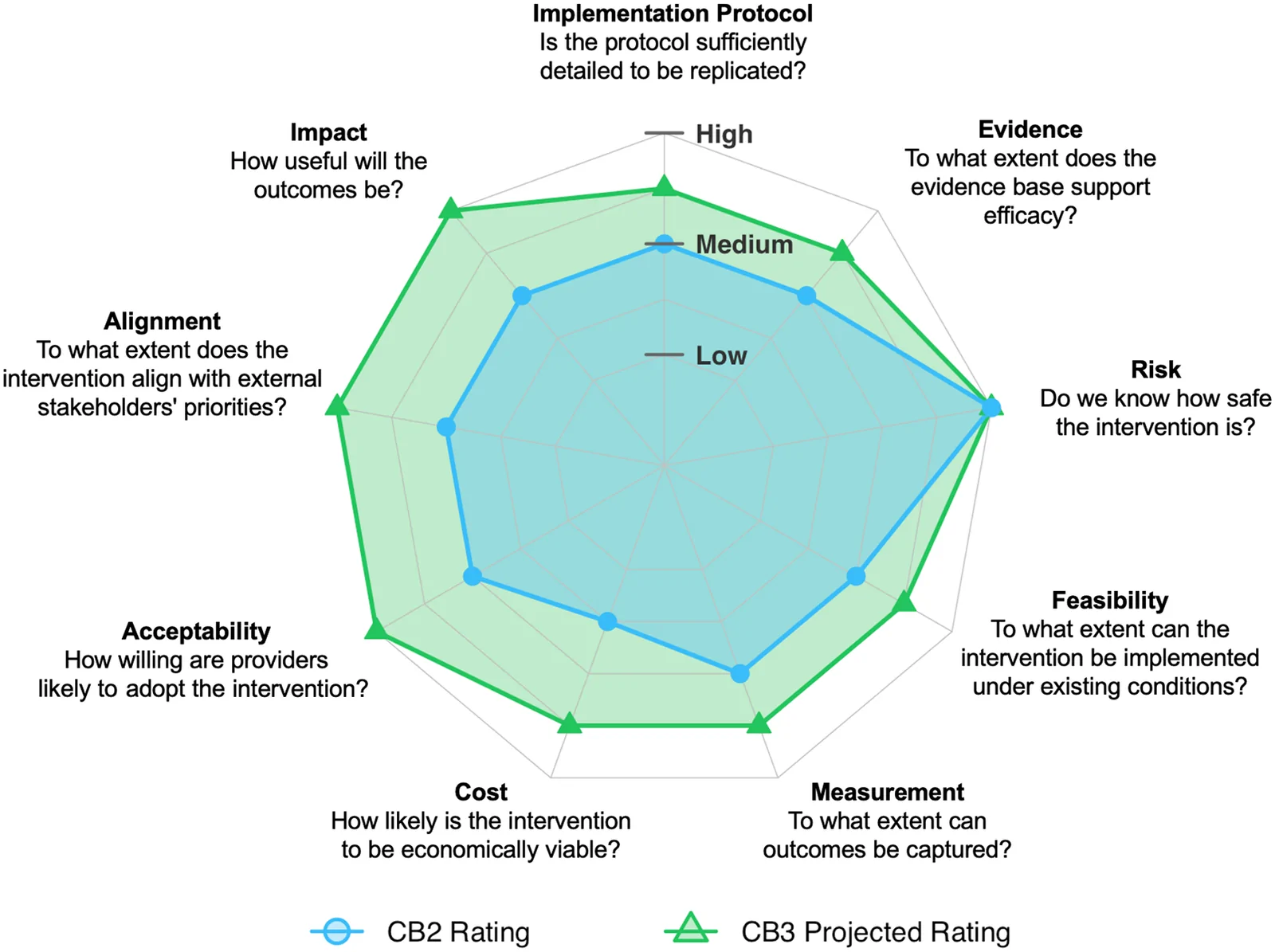
Projected improvements in pragmatic trial readiness from CB2 (Phase 2a) to CB3 (Phase 2b) using the Readiness Assessment for Pragmatic Trials (RAPT) model. RAPT domain ratings for CB2 and projected ratings for CB3. Ratings are shown qualitatively (low, medium, high), where higher ratings indicate greater readiness for pragmatic implementation. The figure illustrates relative changes in implementation readiness across domains as the Communication Bridge program progresses. CB2 ratings were assigned qualitatively using published RAPT scoring guidance and reported trial characteristics; CB3 ratings reflect planned design modifications intended to improve pragmatic trial readiness and do not represent empirically observed outcomes.

CB3 systematically targets multiple RAPT domains to strengthen pragmatic trial readiness. Evidence and Feasibility are advanced through the evaluation of efficacy and safety in a larger, more diverse cohort, supporting scalability of recruitment and delivery approaches. Planned economic analyses will inform the Cost domain by examining resource use and potential sustainability in community-based care settings. The Measurement domain is further strengthened through exploration of objective indicators of communication participation, including wearable sensor-based approaches, as potential complements to established person-centered outcomes.

To optimize Acceptability and Alignment, CB3 incorporates structured stakeholder input from individuals with PPA, communication partners, and interdisciplinary advisors to ensure that intervention design and delivery align with patient priorities and health care system contexts. Finally, CB3 includes planned responder analyses to better characterize variability in treatment response, informing future efforts to tailor intervention delivery and enhance real-world implementation. Similar analyses in CB2 suggested broad applicability across PPA subtypes, supporting the value of responder analysis as an implementation-focused strategy. Collectively, these strategies are intended to advance CB3 toward medium-to-high readiness across RAPT domains and prepare the intervention for subsequent pragmatic evaluation.

## Learning Opportunities From Early-Phase CB Trials

Experiences from the CB1 and CB2 trials yielded several learning opportunities relevant to the development of behavioral interventions for rare, progressive neurodegenerative conditions, while also highlighting persistent questions that require continued investigation.

### Trial Design and Recruitment

The relative rarity of PPA, combined with wide geographic dispersion, limited the feasibility of traditional, multisite-based recruitment strategies. Telehealth delivery expanded access to individuals and families who would otherwise be unable to participate and allowed for a single enrollment site with centralized training of clinicians. From the participant enrollment perspective, the technology-based intervention functioned as both a facilitator and a constraint for some individuals. While videoconferencing platforms enabled multimodal communication support and reduced travel burden, participants and communication partners varied in familiarity with digital tools. These findings underscore the importance of anticipating technological variability and establishing screening and intervention protocols that balance flexibility with minimum technology competencies required for meaningful participation. The collective network of referral support from clinicians, nongovernmental advocacy groups, and targeted outreach efforts were key collaborations that helped our trial reach its enrollment goals. These experiences highlight the need for flexible recruitment approaches, anticipating technological variability, and proactive support strategies when designing remote interventions for underserved and geographically dispersed populations. Technology readiness, which was considered at screening, as well as participant and communication-partner engagement, which were monitored throughout the trials, were important factors influencing trial participation and intervention delivery. In addition, support for strategy use beyond scheduled sessions was a core therapeutic aim, as the intervention was designed to promote enactment of individualized communication strategies in everyday life in alignment with participants' goals.

The current trials were intentionally limited to English-speaking participants and individuals with mild-to-moderate PPA during early-phase intervention development, which necessarily constrains generalizability. Future work will need to address linguistic diversity, multilingual materials, clinician language capacity, technology access and digital literacy, and adaptation for more advanced stages of disease as important implementation and equity priorities. Some of these efforts, including collaborative work related to adaptation and broader access, are already underway.

### Fidelity and Clinical Flexibility

Clinician training and intervention fidelity represented another key learning area. Delivering a standardized intervention in the context of a progressive neurodegenerative disorder required clinicians to balance adherence to core intervention components with clinical judgment and responsiveness to changing participant needs. Early-phase trials highlighted that fidelity in behavioral interventions does not necessitate rigid uniformity, but rather consistency in therapeutic principles and goals. Thus, precision and tailored interventions are possible without compromising rigor or limiting opportunities for broader implementation. This insight has important implications for scaling behavioral interventions while preserving clinical flexibility and has been increasingly recognized in implementation science frameworks for complex, multicomponent behavioral interventions (Bellg et al., 2004; Carroll et al., 2007). The NIH Behavior Change Consortium has established that treatment fidelity encompasses multiple domains including study design, training, intervention delivery, receipt, and enactment, which allows for clinical flexibility while preserving the integrity of core intervention components (Bellg et al., 2004). In CB1, clinician training and adherence to core intervention principles were emphasized, but formal external fidelity assessment was not a component of the pilot trial. In CB2, fidelity assessment was expanded and evaluated from multiple vantage points, reflecting the increased methodological rigor of a later-stage efficacy trial and aligning more closely with standards commonly expected in pharmacologic research. This multidimensional approach to fidelity assessment has been retained in CB3. Across trials, core elements such as the dyadic format, individualized goal-based intervention structure, and overall treatment framework remained consistent, while clinicians exercised structured flexibility in the pacing, examples, and strategy emphasis used to support participant-specific communication participation goals. Fidelity monitoring and treatment differentiation procedures in CB2 and CB3 helped preserve separation between the experimental and control conditions while allowing clinically appropriate adaptation within the intervention.

### Person-Centered Outcome Measurement

Outcome measurement required sustained thoughtfulness throughout the early trial phases. Traditional impairment-based language assessments were often insufficient to capture changes that participants and families perceived as meaningful. At the same time, person-centered and impairment-based approaches are not mutually exclusive. In the CB trials, the distinction was not intended as a strict dichotomy, but rather as a difference in the relative emphasis placed on impairment-focused skill development versus participation-focused communication support, as well as the level at which outcomes were defined and evaluated. The intervention was organized around individualized communication-participation goals, while still permitting use of impairment-based strategies when clinically appropriate to support those broader goals. Consistent with this framework, the theoretical fidelity model evaluated intervention sessions along a continuum, with one end reflecting greater impairment-based emphasis and the other greater participation-based emphasis.

Experiences from CB1 and CB2 reinforced the importance of person-centered outcome measures capable of detecting improvement, stability, or slowed decline in functionally relevant domains. Validated tools specifically designed for PPA remain largely absent; accordingly, parallel efforts have focused on advancing outcome measurement in this population (Fegter et al., 2025; Roberts et al., 2025). More broadly, the challenge of defining and measuring meaningful change in progressive neurodegenerative conditions has been widely recognized, with growing consensus that measures optimized for detecting rapid change may lack sensitivity for conditions in which progression unfolds over years rather than months (Fegter et al., 2025; Gallée et al., 2023; Khayum et al., 2012; Rogalski & Khayum, 2018; Rogalski et al., 2016).

### Retention and Longitudinal Engagement

Finally, retention and longitudinal engagement emerged as critical considerations. Progressive decline, evolving caregiver burden, and competing life demands affected continued participation over time. Sustained engagement was facilitated by interventions that prioritized relevance, flexibility, and dyadic, relationship-based care. These observations emphasize the importance of designing behavioral trials that account for the realities of long disease trajectories and support ongoing participation by both individuals with PPA and their communication partners.

## Discussion

The CB research program provides an exemplar of systematic intervention development, which has been grounded in partnership with individuals with lived experience of PPA and their families from inception. This collaborative approach has shaped intervention targets, delivery methods, and outcome selection, while acknowledging the inherent challenges of intervention development in a rare, heterogeneous, and progressive neurodegenerative syndrome. For speech-language pathologists (SLPs), this work underscores the value of rigorous, staged intervention development, the legitimacy of maintenance as a meaningful outcome in progressive conditions, and the central role of SLPs in advancing both efficacy and implementation of nonpharmacologic care.

The programmatic approach for CB was intentionally designed to support rigor, reproducibility, and eventual scalability. The outcomes and trial elements align with the expectations of a pharmacologic trial. The Stage Model provided helpful guidance for intervention evolution and maturation, which strengthened the evidence base for these nonpharmacologic interventions. Application of the Stage Model with integration of RAPT or similar tools more broadly may help guide the development of future rigorous interventions. Although the pace of clinical trials in PPA has necessarily been deliberate, it has notably required more than a decade of development and testing, reflecting the labor-intensive, resource-heavy nature of this work. Identification of opportunities for efficiencies while maintaining rigor is a key priority for enabling more expedient movement from conception to implementation.

Together, the staged progression of the CB trials illustrates the distinct role of efficacy trials in advancing behavioral intervention science. Early-phase and single-case designs were essential for establishing feasibility and refining intervention components, particularly in a rare and heterogeneous syndrome such as PPA. In contrast, RCTs provided necessary evidence that the intervention could produce meaningful benefit under clearly defined conditions. Importantly, efficacy established through RCTs reflects outcomes under specific trial parameters, including inclusion criteria, intervention dose, delivery format, and outcome measurement, and does not determine effectiveness across all clinical contexts or populations. Accordingly, generalization beyond trial conditions should proceed cautiously and be guided by empirical evidence examining how individual characteristics, disease stage, and environmental context modify intervention response. Within the CB program, early efficacy trials provide a foundation for subsequent work aimed at refining appropriate use across several “wh” domains, including *who* benefits most, *when* intervention should be initiated, *what* duration is optimal, and *where* intervention delivery refinements can maximize impact, feasibility, and cost efficiency.

The findings from the CB trials underscore the importance of person-centered outcome measurement in intervention research for PPA and related dementias. In CB2, individuals in the experimental arm demonstrated improvement and maintenance of personalized communication goals over 12 months, an outcome that is clinically meaningful in the context of a progressive neurodegenerative syndrome. Maintenance of functional abilities over this time frame is particularly salient for individuals with PPA, for whom communication impairment can impact social participation, relationships, autonomy, functional activities of daily living, and quality of life (Moeller et al., 2021). These results highlight how behavioral interventions can meaningfully alter lived experience even when underlying neuropathology continues to evolve.

Beyond clinical outcomes, the CB model may also help reduce practical burdens associated with specialty care for PPA, particularly through telehealth delivery. By reducing travel demands, transportation barriers, time away from work, and scheduling disruptions for both individuals with PPA and their communication partners, remote intervention may lessen some of the indirect costs associated with ongoing care. Telehealth delivery may also support continuity of care by allowing participants to continue sessions while traveling or after relocation. These burden-related considerations are relevant to the planned economic analyses in CB3 and may help clarify the broader value of this model beyond clinical outcomes alone.

An additional implementation consideration is the current status of the CB web application. At present, CB remains a research-based intervention infrastructure and is not commercially available. Importantly, it is designed for use with the guidance of a trained speech-language pathologist rather than as a standalone patient-facing digital tool. Future dissemination pathways will need to address platform access, clinician training, infrastructure requirements, and sustainable approaches to broader clinical adoption.

CB trial outcomes should be interpreted within the broader therapeutic landscape of Alzheimer's disease and related dementias. Recent pharmacologic trials have demonstrated modest attenuation of decline on global cognitive and functional measures, with participants continuing to worsen over time despite treatment (Sims et al., 2023; van Dyck et al., 2023). In contrast, the CB intervention targets functional communication and participation—domains that are not well captured by traditional impairment-based measures. Although these approaches assess fundamentally different constructs, the contrasting outcome trajectories emphasize the complementary roles of disease-modifying therapies and person-centered behavioral interventions. Together, they suggest that comprehensive care for individuals with PPA may require integrated approaches that address both biological disease processes and the functional, relational, and psychosocial dimensions of living with a progressive communication disorder.

## Ethics Statement

All trials referenced were conducted in accordance with institutional ethical standards and approved by the appropriate institutional review boards (IRBs). Because this manuscript is a review article and does not involve new human participant research, IRB approval and informed consent are not applicable; therefore, no IRB name, project number/ID, or informed consent statement has been included.

## Data Availability Statement

No new data were generated or analyzed in this review. All data referenced are available in the published literature or through the original trial publications.

## Artificial Intelligence Statement

ChatGPT was used during manuscript editing to enhance grammar and readability. All suggestions were reviewed and revised by the authors before inclusion. Because artificial intelligence was used only for language editing and not for content generation, parenthetical citation was not applied. All authors assume responsibility for the accuracy, originality, and integrity of all text in the review article.
